# Author Correction: Jungle Express is a versatile repressor system for tight transcriptional control

**DOI:** 10.1038/s41467-018-07111-2

**Published:** 2018-10-30

**Authors:** Thomas L. Ruegg, Jose H. Pereira, Joseph C. Chen, Andy DeGiovanni, Pavel Novichkov, Vivek K. Mutalik, Giovani P. Tomaleri, Steven W. Singer, Nathan J. Hillson, Blake A. Simmons, Paul D. Adams, Michael P. Thelen

**Affiliations:** 1 Joint BioEnergy Institute, Emeryville, CA 94608 USA; 2 Institute of Botany, University of Basel, 4001 Basel, Switzerland; 3 Molecular Biophysics and Integrated Bioimaging Division, Lawrence Berkeley National Laboratory, Berkeley, CA 94720 USA; 4 Department of Biology, San Francisco State University, San Francisco, CA 94132 USA; 5 Environmental Genomics and Systems Biology Division, Lawrence Berkeley National Laboratory, Berkeley, CA 94720 USA; 6 Biological Systems and Engineering Division, Lawrence Berkeley National Laboratory, Berkeley, CA 94720 USA; 7 Department of Bioengineering, University of California Berkeley, Berkeley, CA 94720 USA; 8 Biology and Biotechnology Division, Lawrence Livermore National Laboratory, Livermore, CA 94550 USA

Correction to: *Nature Communications*; 10.1038/s41467-018-05857-3, published online 06 September 2018

In the original version of this Article, an incorrect URL was provided in the Data Availability Statement regarding the deposition of plasmids listed in Supplementary Table 4. The correct URL is https://public-registry.jbei.org/folders/378. This error has been corrected in both the PDF and HTML versions of the Article.

