# Supplementary material for: Jungle Express is a versatile repressor system for tight transcriptional control
Source: Nat Commun. 2018 Sep 6;9:3617. doi: 10.1038/s41467-018-05857-3 (PMC6127294; doi:10.1038/s41467-018-05857-3)
Supplement: Supplementary file 1 — Supplementary Information [file 41467_2018_5857_MOESM1_ESM.pdf]

Supplementary Information

Ruegg *et al.*

Jungle Express is a versatile repressor system for tight transcriptional control

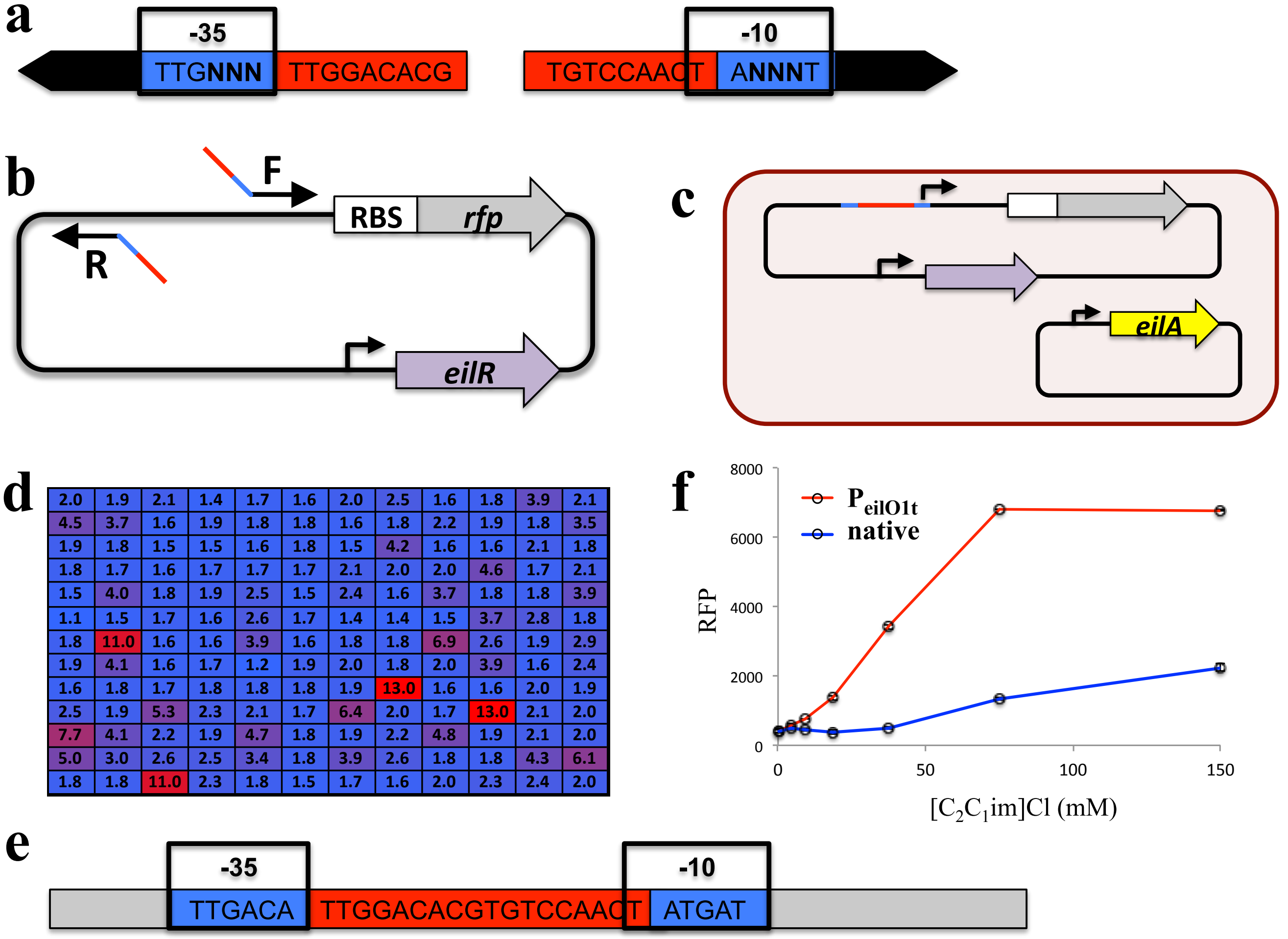

Supplementary Figure 1 | Construction of the EilR-regulated reporter plasmid. (a) Primers used to build a promoter library with randomized base pairs (N) in the -35 and -10 promoter hexamer regions (boxed). The consensus *eil*-operator (red) was truncated to fit into a typical *E. coli* 17-bp spacer between promoter hexamers and overlapped by 1 bp with the -10 hexamer. Each oligonucleotide contained 9 base pairs of the truncated *eilOc*. **(b)** Plasmid pFAB-eilR containing *rfp* (gray) and *eilR* (purple) that is driven from a weak constitutive promoter, was PCR-amplified with randomized oligonucleotides annealing at a region upstream of the *rfp* ribosome binding site (white). **(c)** The PCR products were circularized and transformed into *E. coli* cells containing plasmid pBbS5c-*eilA* that expresses the EilA efflux pump from the independently regulated P_{lacUV5} to provide tolerance to the ionic liquid $[C_2C_1im]Cl$ during the subsequent library screening. **(d)** Colonies (136) were picked and grown in LB media (Kan/Cm) with (ON) and without 300 mM $[C_2C_1im]Cl$ (OFF). RFP fluorescence was measured in a spectrophotometer (584nm/607nm), and inducibility of variants with ON/OFF ratios higher than 10 was confirmed. **(e)** The resulting promoter variant with the highest dynamic range, termed P_{EilO1t}, was selected. **(f)** Fluorescence of *E. coli* expressing RFP from either the native intergenic regulatory region (blue) or from P_{EilO1t} in the presence of $[C_2C_1im]Cl$. Values and error bars represent the means and standard deviation of measurements from two independently grown cultures.

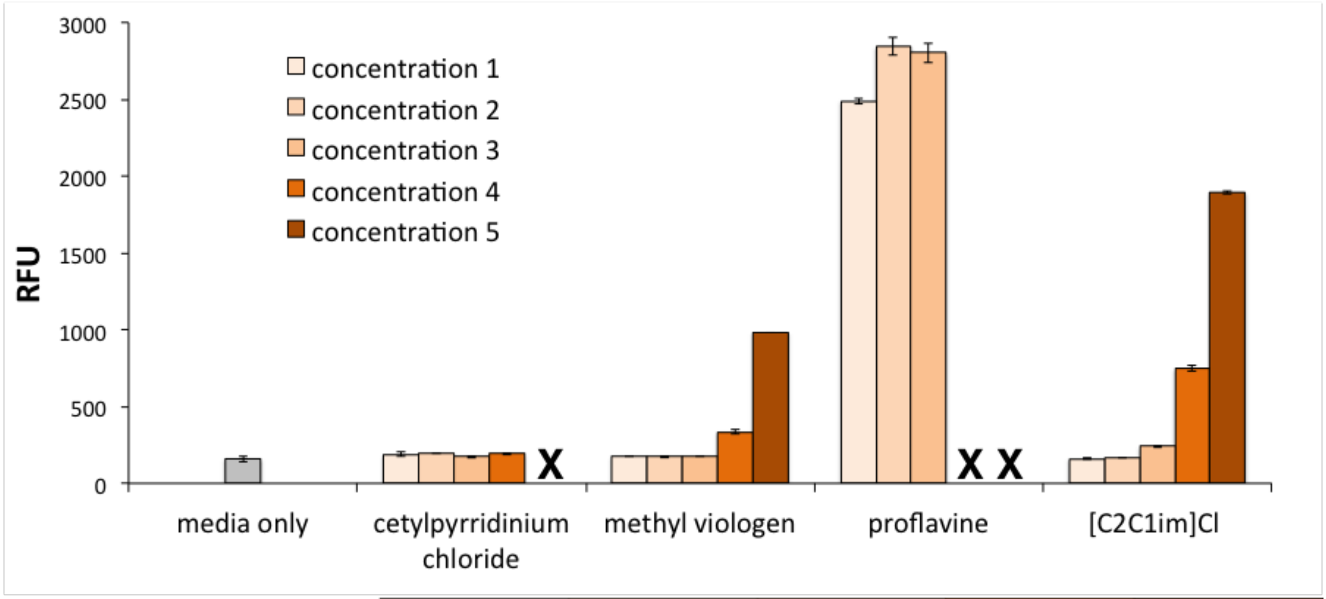

	concentration 1	concentration 2	concentration 3	concentration 4	concentration 5
cetylpyrridinium chloride (μM)	1.25	2.5	5	10	20
methyl viologen (μM)	12.5	25	50	100	200
proflavine (μM)	12.5	25	50	100	200
$[C_2C_1im]Cl$ (mM)	0.16	0.8	4	20	100

Supplementary Figure 2 | Response of the P_{EilO1t}-reporter strain to substrates of the EilR-regulated efflux pump EilA[1]. Normalized fluorescence (in relative fluorescence units, or RFU) of stationary phase *E. coli* expressing RFP from P_{EilO1t}. Cells were grown in the presence of EilA substrates at different concentrations. "X" indicates the absence of observable growth. Values and error bars represent the means and standard deviation of measurements from two independently grown cultures.

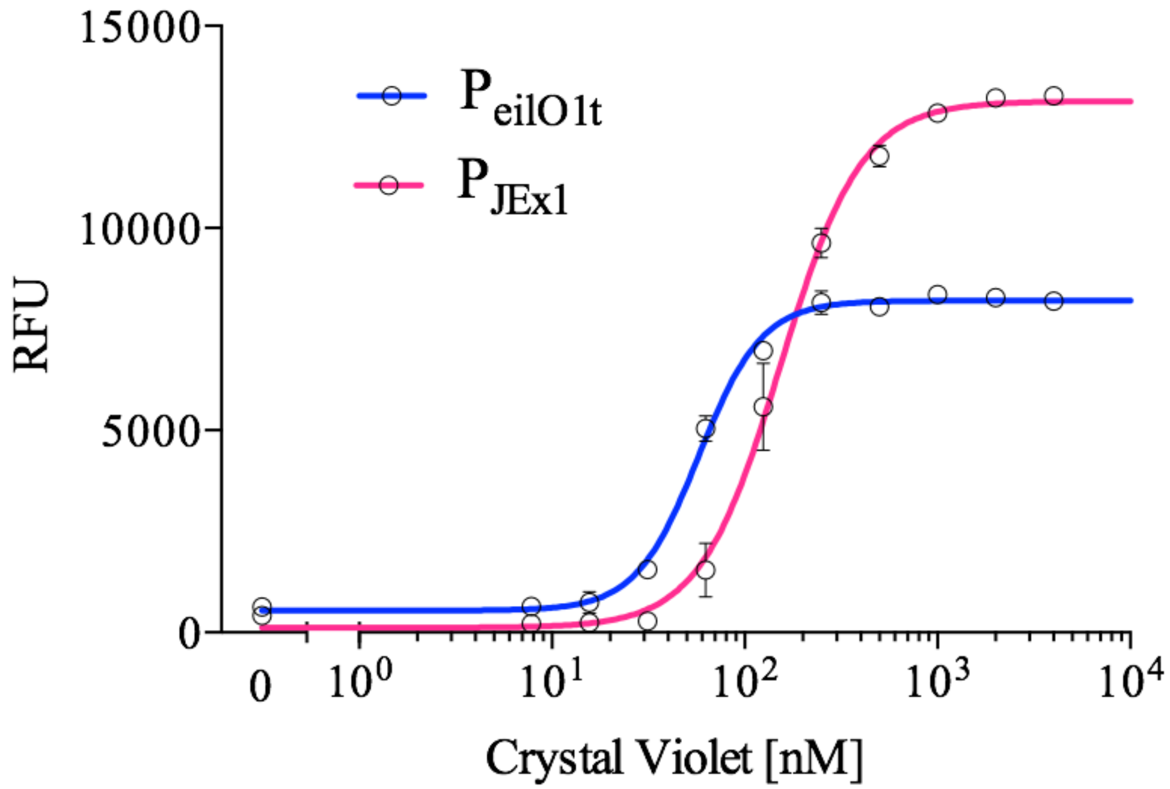

Supplementary Figure 3 | Addition of a full-length operator increases the dynamic range. Normalized fluorescence in relative fluorescence units (RFU) of *E. coli* expressing RFP from the EilR-regulated promoters containing either a truncated operator *eilOt* between the -10 and -35 sites only (P_{eilO1t}, blue) or an additional full-length consensus operator *eilOc* that coincides with the transcriptional start (P_{JEx1}, pink). Maximal induction by crystal violet was reached at ~0.2µM and 1µM, respectively. Measurements were taken after cells were grown to stationary phase in EZ-Rich defined medium containing 0.2% glucose. Values and error bars represent the means and standard deviation of measurements from three independently grown cultures expressing RFP from plasmids pTR_eilO1t and pTR_aJEx1-rfp.

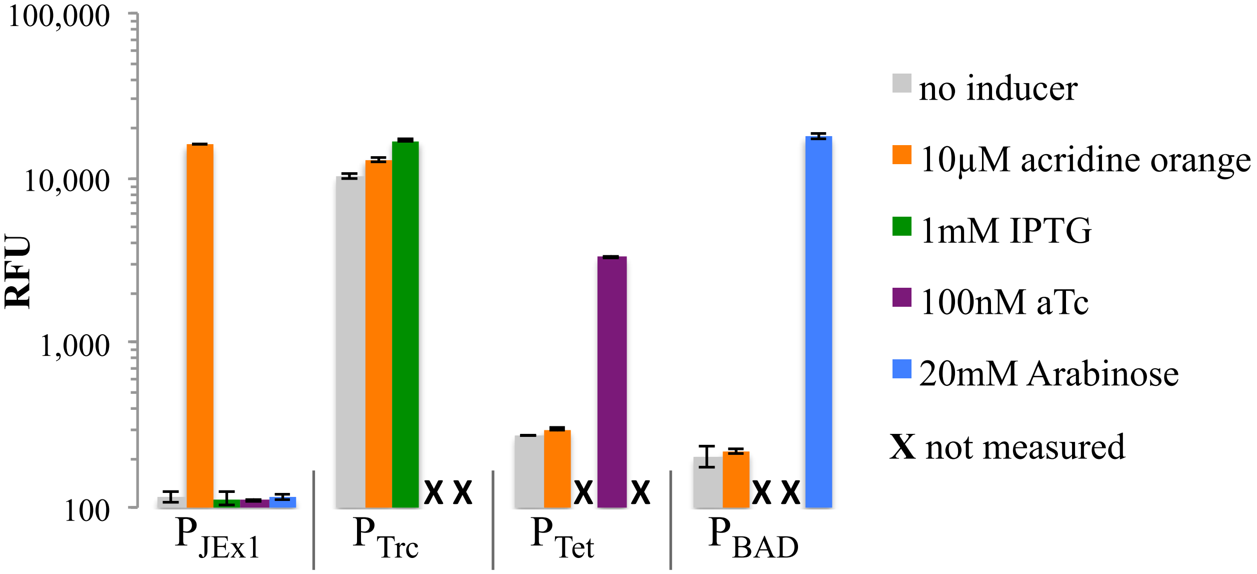

Supplementary Figure 4 | Comparison of inducer specificities with respect to their corresponding promoters. Relative fluorescence units (RFU) of *E. coli* cells expressing RFP from the EilR-regulated P_{JEx1} promoter, the LacI-regulated P_{trc}, the TetR-regulated P_{tet}, and the AraC-regulated P_{BAD} promoters[2], which were induced with the corresponding effector molecules, acridine orange, isopropyl β-D-1-thiogalactopyranoside (IPTG), anhydrotetracycline (aTc), or arabinose (Ara). Cultures were induced at early log phase and grown to stationary phase in terrific broth for 21 hours. *E. coli* background fluorescence was not deducted in this figure. Values and error bars represent the means and standard deviation of measurements from three independently grown cultures.

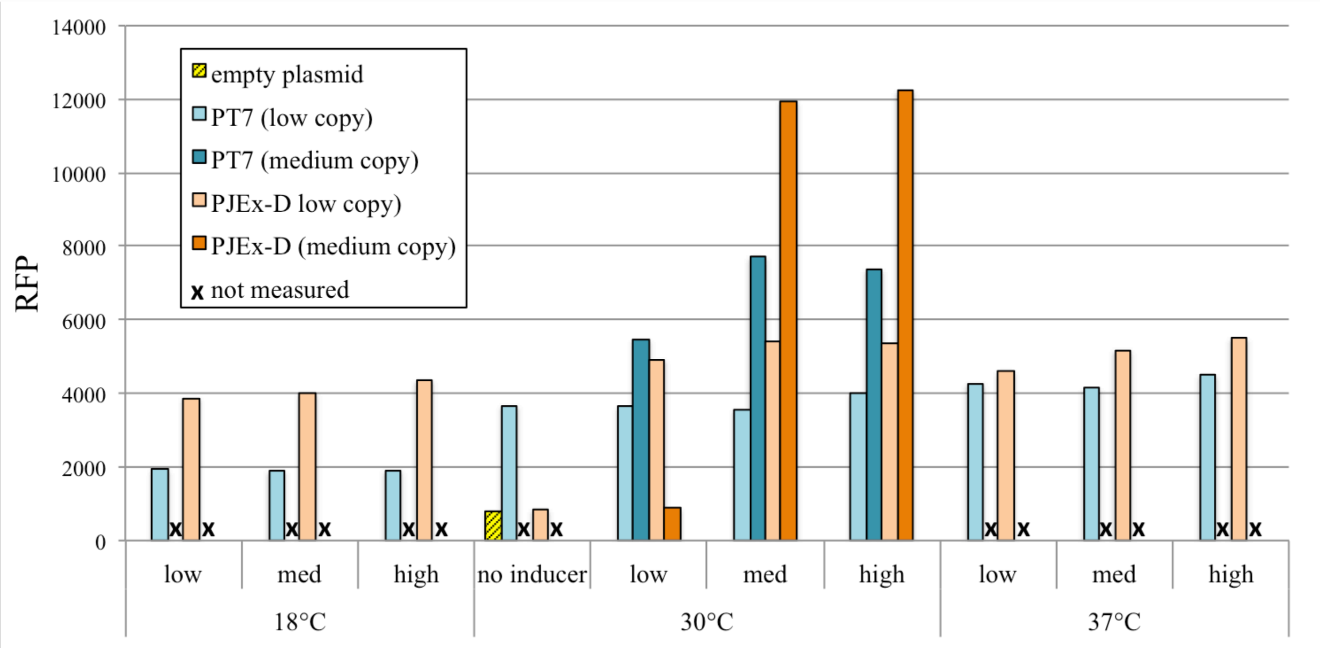

Supplementary Figure 5 | RFP expression from P_{JExD} and P_{T7}. *E. coli* BL21(DE3) expressing RFP either driven from the EilR-regulated P_{JExD} (orange) or from a LacI-regulated P_{T7}[2] (blue), located either on low-copy plasmids (pSC101 *ori*, light color) or on medium-copy plasmids (p15A *ori*, dark color). Cell cultures grown in 5 mL terrific broth were induced at early logarithmic phase with the corresponding inducers, crystal violet or IPTG, for protein expression at three temperatures: 18 °C for 120 h, 30 °C for 50 h and 37 °C for 50 h. Crystal violet concentrations for inducing the P_{JExD} system ranged from 250 nM (low), 500 nM (med) to 1000 nM (high), while IPTG levels for the LacI-regulated T7 system were 250 µM (low), 500 µM (med) and 1 mM (high). Fluorescence measurements after growth at 30°C were also taken from uninduced cultures containing the low-copy plasmid version as well as from a control strain lacking *rfp* (yellow). Values represent measurements of single cultures.

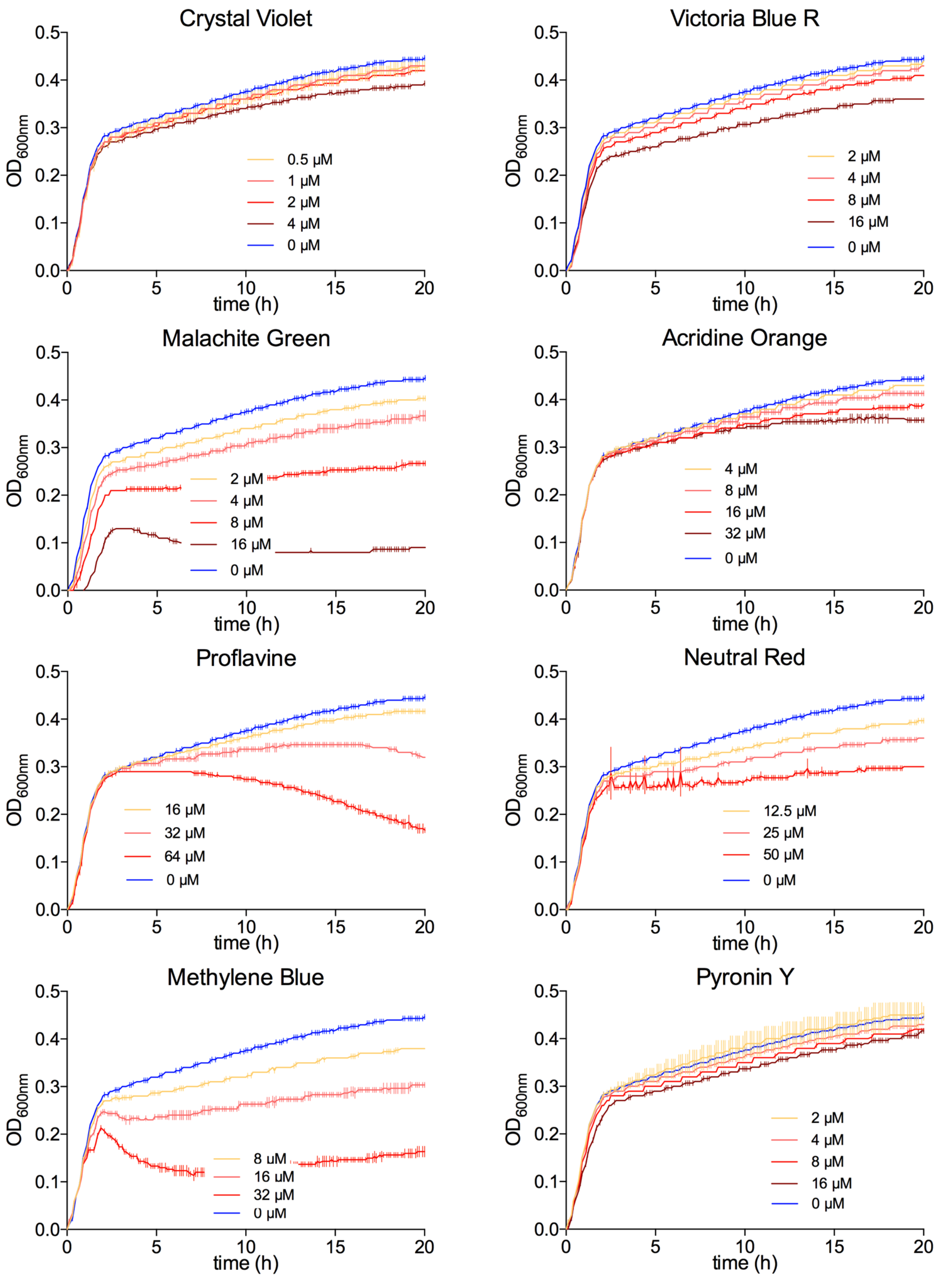

Supplementary Figure 6 | Growth characteristics of *E. coli* in response to inducing dyes. *E. coli* DH10B containing a control plasmid (pSC101 *ori*; Km^R) were grown at 37 °C in LB containing kanamycin (50 µg/mL) to early exponential phase, when dyes were added at concentrations that were equal to or higher than levels required for full induction. OD_{600} measurements were performed after addition of dyes in a BioTek Synergy 2 instrument; values were normalized to the OD_{600} at the start of the measurement to account for intrinsic dye absorbance at 600 nM. Values and error bars represent the means and standard deviation of measurements from three independently grown cultures.

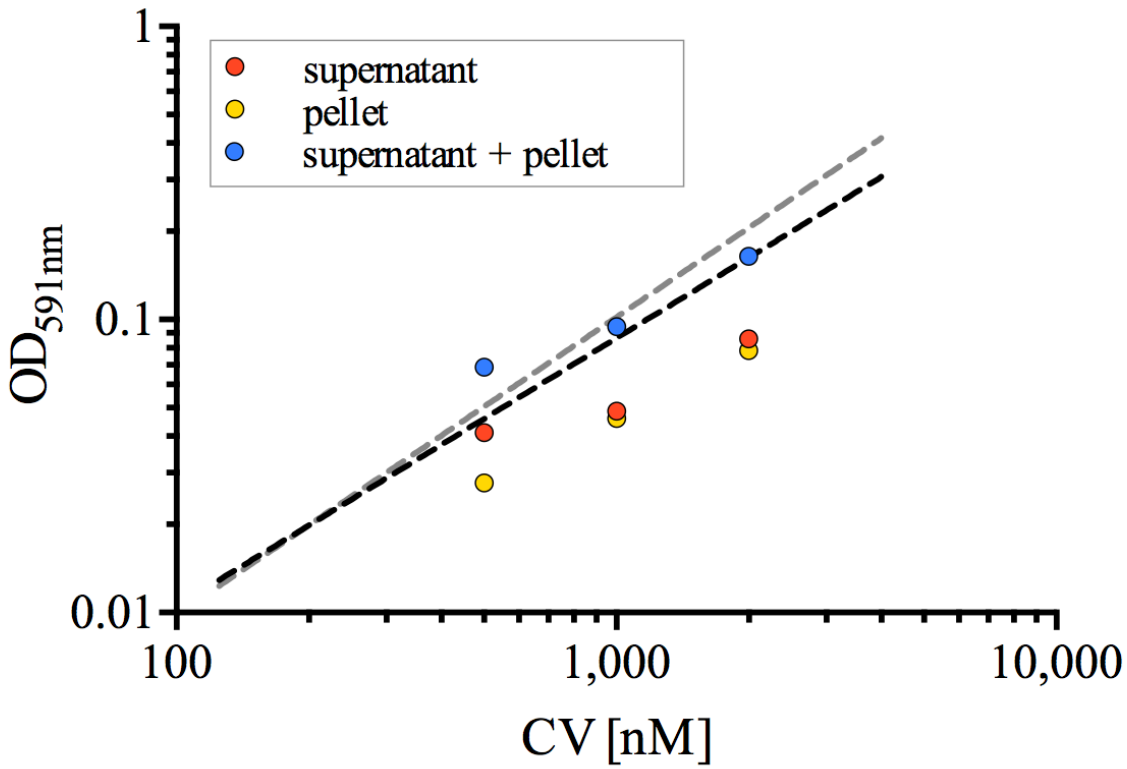

Supplementary Figure 7 | Stability of crystal violet in culture medium. *E. coli* DH10B was grown in 5 mL LB containing 0.5, 1 or 2 µM crystal violet (CV). After 15 hours of growth at 37 °C, 1.8 mL of the cultures were centrifuged at 15,000 rpm for one minute. The media supernatant was transferred to 1 mL cuvettes and cell pellets were resuspended in 600 µL 1:1 acetone/ethanol for CV extraction. After 2 minutes centrifugation at 15,000 rpm, the organic supernatant was transferred to cuvettes containing 1200 µL water. Crystal violet concentration was measured with a spectrophotometer at 591 nm, the wavelength of maximal absorption of this dye. Data points represent the average measurements of two independently grown cultures. Error bars indicating the standard deviations are not visible due to small variations. Standard curves were generated by linear regression of six absorbance log values of a two-fold CV dilution series ranging from 0.125 µM to 4 µM in both LB (black line) and the solution used to measure CV extracted from the cell pellets (gray line). R-square values for standard curves were 0.9910 (LB), and 0.9999 (extraction solution).

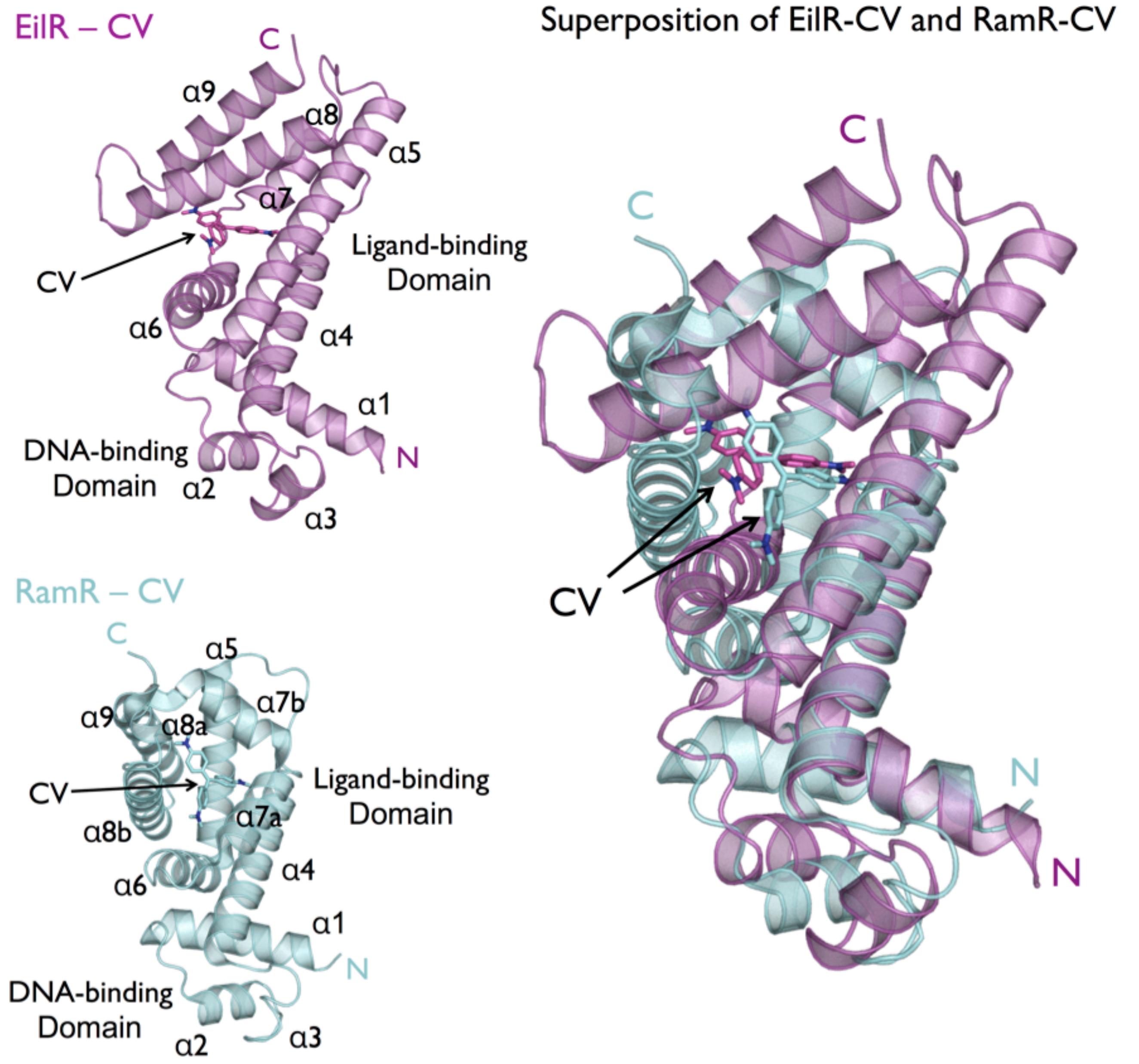

Supplementary Figure 8 | Comparison of the CV binding sites in EilR and RamR. EilR-CV (upper left) and RamR-CV (PDB ID 3VW1[3]; lower left) monomeric domains are superpositioned (right), indicating that the CV molecule is bound to the core of the ligand-binding domain in both EilR and RamR structures. A significant shift between the CV binding sites is observed as a result of differences in the surrounding protein fold. The major differences between EilR and RamR are observed in the orientation of α7 and α8 in EilR and the corresponding subdivided helices α7a, α7b, α8a and α8b in RamR.

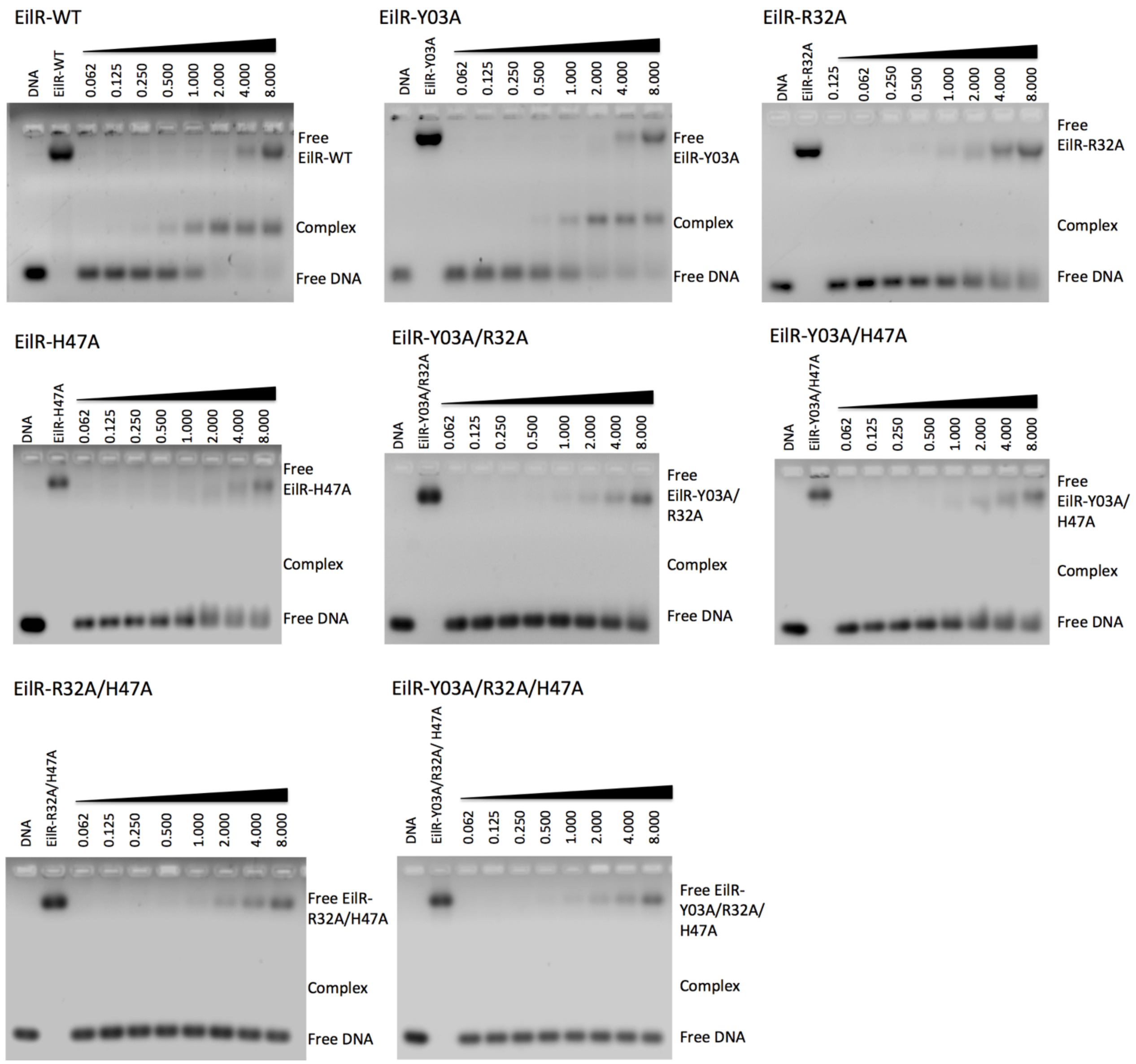

Supplementary Figure 9 | Comparisons of wild-type EilR and mutants with Ala substitutions of residues involved in base pair recognition of the *eilO* operator. Purified wild-type EilR (WT) and derivatives mutated in the DNA-binding domain were assessed by EMSA after adding the duplexed oligonucleotide 5'-AAAAAGTTGGACACGTGTCCAACTTTCC-3' (*eilOc* operator underlined). Molar ratios of protein to DNA from 0.062:1 to 8:1 are indicated on the top of the gels. Tyr3, Arg32 and His47 were mutated to Ala. The single mutant EilR-Y03A protein had a decreased affinity for the *eilO* operator with the protein-DNA complex beginning to appear in the lane with a 0.5:1.0 ratio (EilR-Y03A:DNA), while the wild-type protein formed a complex in the lane with a 0.125:1 ratio (EilR-WT:DNA). The single mutations R32A and H47A completely impaired EilR binding to *eilO*. No protein-DNA complexes were observed for the mutants EilR-R32A, EilR-H47A, EilR-Y03A/R32A, EilR-Y03A/H47A, EilR-R32A/H47A and EilR-Y03A/R32A/H47A.

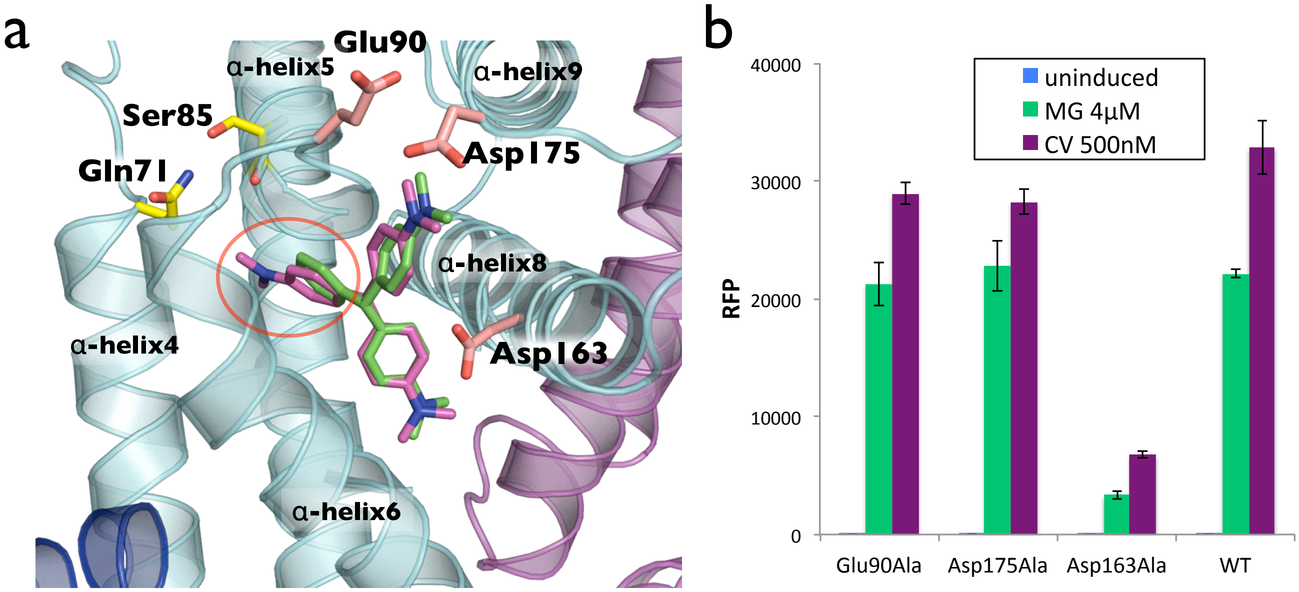

Supplementary Figure 10 | Additional contacts of CV and MG in the ligand binding pocket and substitution of negatively charged residues. (a) X-ray crystallographic analysis showing the ligand-binding site of EilR interacting with MG (green) and CV (purple). Superposition of EilR-MG and EilR-CV structures highlights residues Gln71 in α-helix4 and Ser85 in α-helix5 (yellow) that establish additional van der Waals contacts by the extra dimethyl amino group of CV (red circle). Residues Met67, Ala86, and Trp125 (not shown here, see Fig. 4) interact with both inducers, each via one (MG) or two (CV) contacts. Negatively charged residues Glu90, Asp163 and Asp175 that were mutated in (b) are shown in pink. **(b)** Fluorescence of *E. coli* cultures expressing P_{JExD}-driven RFP and regulated by either the wild-type EilR (WT) or mutants with Ala substitutions of negatively charged residues. Mutations were prepared from plasmid pTR_aJExD-rfp. Measurements were taken after cells were grown to stationary phase in LB medium. Values and error bars represent the means and standard deviations of measurements from two independently grown cultures.

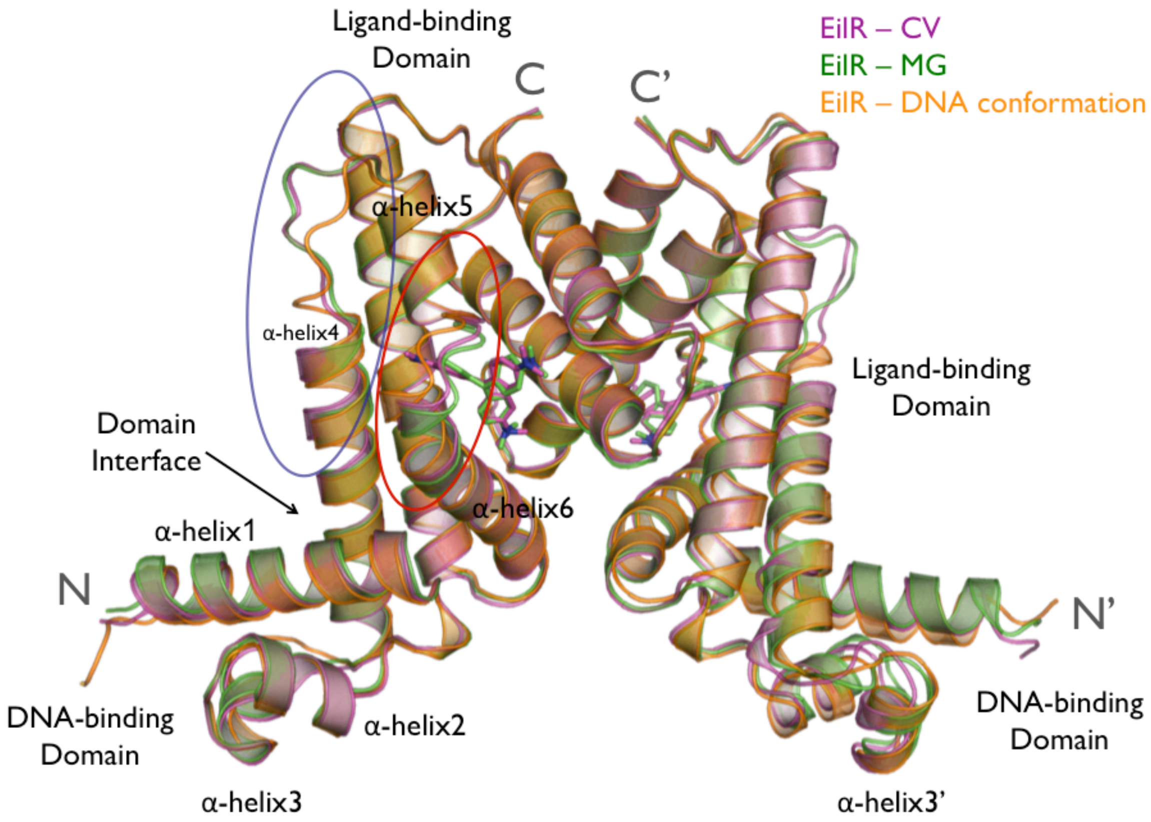

Supplementary Figure 11 | Influence of CV and MG binding on DNA binding domains. Superposition of the EilR dimer in the CV and MG ligand-bound forms (magenta and green, respectively) and DNA-bound form (orange) showed a significant conformational change around the inducer binding site. The binding of the inducers CV and MG was coupled with a shift in the loops connecting α-helix4/α-helix5 (blue circle) and α-helix5/α-helix6 (red circle). These changes have an impact on α-helix4 and α-helix6, which are located at the interface between the DNA-binding and the inducer-binding domains. Consequently, a slight difference in the distance between the DNA-binding domains of the EilR dimers of the induced state and the DNA-bound state was observed, suggesting a possible cascade mechanism for the induction signal to be transferred to the DNA-binding domain. The EilR-CV and EilR-MG complexes showed an increased distance between the DNA binding domain α-helix3 and α-helix3' of 0.7 Å and 1.8 Å, respectively, when compared to the DNA-bound state.

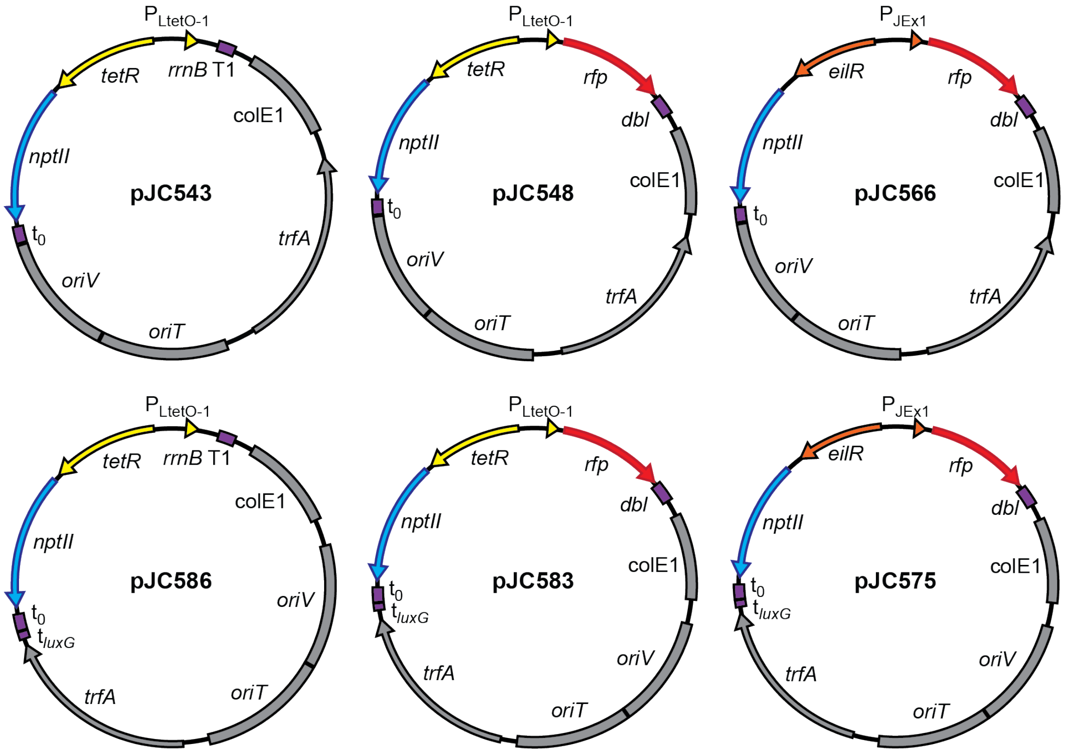

Supplementary Figure 12 | Representative maps of plasmids used for promoter assays in *P. putida, C. crescentus, and S. meliloti*

Supplementary Table 1 | *E. coli* and *S. meliloti* mutant frequencies in the presence of CV. *E. coli* strain BW25139 was used to determine the frequency of rifampin-resistant (Rif^R) mutants, while *S. meliloti* strain JOE3608 was used to determine the frequency of mutants that do not require taurine for growth.

Treatment	*E. coli* BW25139 [a] $f(10^{-9})$ Rif^R	*S. meliloti* JOE3608 [b] $f(10^{-6})$ taurine independence
Medium	3.8 (± 1.8)	7.0 (± 0.4)
Medium + 1 μM CV	8.6 (± 2.3)	8.3 (± 1.1)
1% EMS	1530 (± 630)	N/A

[a] Previously published procedures[4,5] were followed to assess the frequencies of Rif^R mutants in *E. coli* strain BW25139 (gift of Barry L. Wanner; *recA*$^+$ progenitor of BW25141[6]) and, for comparison, to treat cells with ethyl methanesulfonate (EMS), an agent commonly used for mutagenesis. Briefly, approximately 1000 cells were used to seed 0.4 mL of LB culture, grown overnight with aeration for 20 hours to stationary phase, and plated onto LB and LB containing 50 μg mL^{-1} rifampin. For the CV treatment, cultures were grown with 1 μM CV overnight. For EMS mutagenesis, cells were first grown to mid-logarithmic phase in LB, washed and resuspended in cold minimal A buffer [10.5 g K_2HPO_4, 4.5 g KH_2PO_4, 1 g $(NH_4)_2SO_4$, 0.5 g sodium citrate·$2H_2O$ per liter], mixed with EMS to a final concentration of 1%, and shaken at 37°C for 30 minutes; cells were then washed in minimal A buffer and resuspended in LB, and 0.1 mL of a 10^{-3} dilution was added to 0.3 mL of LB and grown overnight for subsequent plating. Mutant frequency for each culture was determined by dividing the number of Rif^R mutants by the colony forming units (CFU) on LB. The mean (± standard error of the mean) of each treatment was calculated using a total of eight independent cultures (two cultures only for EMS treatment).

[b] Mutant frequency in *S. meliloti* was assessed using strain JOE3608 [Δ*pleC*::Ω / pJC476 (P_{tau}-*pleC*)][7], which requires taurine for growth because the only copy of an essential gene, *pleC*, is under the control of a taurine-dependent promoter on a plasmid. Approximately 10^6 cells per mL were grown overnight with aeration for 20 hours in 3 – 4 mL PYE medium containing 0.5 μg mL^{-1} oxytetracycline and 50 mM taurine, with or without 1 μM CV. Overnight cultures (containing about 10^8 cells per mL) were washed and resuspended in water to an optical density at 600 nm of 0.1, serially diluted, and plated onto PYE containing 1 μg mL^{-1} oxytetracycline, with or without 100 mM taurine. Mutant frequency for each culture was determined by dividing the number of mutants that do not require taurine for growth by the CFU on taurine plates. The mean (± standard error of the mean) of each treatment was calculated using a total of five independent cultures.

Supplementary Table 2 | Concentrations and costs of agents used to induce common regulatable expression systems and EilR-regulated promoters in a 1000 L *E. coli* fermentation (based on lab-scale experiments).

Regulating transcription factor	Inducer	Typical concentration for full induction		Cost per 1000 L (USD) *	Reference
LacI	Isopropyl β-D-1-thio-galactopyranoside (IPTG)	200	µM	1983	[8]
AraC	L-Arabinose	~1-13	mM (0.01-0.2%)	68-1353	[9]
RhaR/RhaS	Rhamnose	12	mM (0.2%)	328	[10]
XylS	*m*-Toluate	2	mM	27	[11]
TetR	Anhydrotetracycline	100	nM	327	[12]
CymR	Cumate	100	µM	104	[13]
EilR	Crystal violet	500	nM	0.08	This work
EilR	Acridine orange	10	µM	14	This work

* Prices are based on the least expensive unit sold at Sigma-Aldrich (January 2018).

Supplementary Table 3 | Summary of crystal parameters, data collection, and refinement statistics. Values in parentheses are for the highest resolution shell.

	EilR-eilO	EilR-MG	EilR-CV
**Crystal parameters**			
Space group	P1	$P2_1$	$P2_1$
Unit-cell parameters (Å)	a = 54.92, b = 81.87, c = 85.46, α =112.57, β = 113.09, γ = 105.94	a = 100.14, b = 85.98, c = 102.27 and β = 101.69	a = 97.39, b = 79.22, c = 105.57 and β = 97.10
**Data collection statistics**			
Wavelength (Å)	0.97965	1.000	1.000
Resolution range (Å)	65.29 – 2.16 (2.20 – 2.16)	50 – 2.90 (2.95 – 2.90)	50 – 3.40 (3.46 – 3.40)
No. of reflections (measured / unique)	180465 (55895)	36167 (1777)	20683 / 915
Completeness (%)	95.7 (96.0)	99.9 (99.8)	93.4 (82.1)
R_{merge}*	0.096 (0.858)	0.138 (0.73)	0.219 (0.73)
Redundancy	3.2 (3.3)	2.9 (3.0)	2.9 (2.8)
Mean I / sigma (I)	8.5 (1.5)	6.8 (1.2)	3.9 (1.6)
CC1/2	0.995 (0.625)	0.995 (0.627)	0.994 (0.876)
**Refinement and model statistics**			
Resolution range (Å)	65.29 - 2.16 (2.20 - 2.16)	48.78 - 2.93 (3.03 - 2.93)	48.64 - 3.40 (3.48 - 3.40)
No. of reflections (work / test)	55872 (2854)	36132 / 2007	20064 / 2019
R_{cryst}§	0.194 (0.309)	0.196 (0.292)	0.204 (0.280)
R_{free}¶	0.228 (0.365)	0.240 (0.324)	0.271 (0.354)
RMSD bonds (Å)	0.002	0.003	0.002
RMSD angles (°)	0.467	0.470	0.491
All-atom Clashscore	2.5	2.6	3.2
Molprobity Score	1.1	1.3	0.93
Rotamer outliers (%)	0	0.2	1.3
B factor (protein / solvent) ($Å^2$)	55.9 / 86.6	56.5 / 53.9	65.18 / 59.7
No. of protein atoms	7156	11976	12000
No. of auxiliary molecules	2 14bp DNA	8 MG	8 CV
**Ramachandran plot**			
Favorable region	97.6	98.1	96.5
Additional allowed region	2.4	1.9	3.4
Disallowed region	0	0	0.07
**PDB ID**	5VL9	5VLG	5VLM

* $R_{merge} = \sum_h \sum_i | I_i(h) - \langle I(h) \rangle | / \sum_h \sum_i I_i(h)$, where $I_i(h)$ is the intensity of an individual measurement of the reflection and <I(h)> is the mean intensity of the reflection.

§ $R_{cryst} = \sum_h ||F_{obs}| - |F_{calc}|| / \sum_h |F_{obs}|$, where F_{obs} and F_{calc} are the observed and calculated structure-factor amplitudes, respectively.

¶ R_{free} was calculated as R_{cryst} using 5.0 % of randomly selected unique reflections that were omitted from the structure refinement.

Supplementary Table 4 | List of plasmids used in this work

Plasmid name	Promoter	Reporter gene	ori	Selection marker	Reference	Part ID
pTR_aJEx1-rfp	P_{JEx1}	*rfp*	p15A	Kan	this work	JBx_092811
pTR_aJExA1-rfp	P_{JExA1}	*rfp*	p15A	Kan	this work	JBx_092812
pTR_aJExA2-rfp	P_{JExA2}	*rfp*	p15A	Kan	this work	JBx_092814
pTR_aJExD-rfp	P_{JExD}	*rfp*	p15A	Kan	this work	JBx_092816
pTR_aJExH1-rfp	P_{JExH1}	*rfp*	p15A	Kan	this work	JBx_092818
pTR_aJExH2-rfp	P_{JExH2}	*rfp*	p15A	Kan	this work	JBx_092819
pTR_aJExL-rfp	P_{JExL}	*rfp*	p15A	Kan	this work	JBx_092820
pTR_sJExA1-rfp	P_{JExA1}	*rfp*	SC101	Kan	this work	JBx_092826
pTR_sJExD-rfp	P_{JExD}	*rfp*	SC101	Kan	this work	JBx_092828
pFAB5088	N/A	*rfp*	p15A	Kan	unpublished*	
pFAB-eilR	N/A	*rfp*	p15A	Kan	this work	JBx_092823
pBbS5c-eilA	P_{lacUV5}	N/A	SC101	Cm	[28]	JBx_023575
pFABeilRig	native P_{eilA}	*rfp*	p15A	Kan	this work	JBx_092824
pFABeilO1t	P_{EilO1t}	*rfp*	p15A	Kan	this work	JBx_092822
pTR_eilO1t	P_{EilO1t}	*rfp*	p15A	Kan	this work	JBx_092810
pBbA0k	Empty control plasmid		p15A	Kan	[52]	JBx_001880
pBbS7k-RFP	P_{T7}	*rfp*	SC101	Kan	[52]	JBp_000054
pBbA7k-RFP	P_{T7}	*rfp*	p15A	Kan	[52]	JBp_000051
pBbA2k-RFP	P_{tet}	*rfp*	p15A	Kan	[52]	JBp_000060
pBbA1k-RFP	P_{trc}	*rfp*	p15A	Kan	[52]	JBp_000069
pBbA8k-RFP	P_{BAD}	*rfp*	p15A	Kan	[52]	JBp_000006
pTR_sJExD-sacB	P_{JExD}	*sacB*	SC101	Kan	this work	JBx_092829
pTR_aJExD-sacB	P_{JExD}	*sacB*	p15A	Kan	this work	JBx_092817
pTR_sJExA1-sacB	P_{JExA1}	*sacB*	SC101	Kan	this work	JBx_092827
pTR_aJExA1-sacB	P_{JExA1}	*sacB*	p15A	Kan	this work	JBx_092813
pTR_aJExD-lacZ	P_{JExD}	*lacZ*	p15A	Kan	this work	JBx_092815
pBbA7k-lacZ	P_{T7}	*lacZ*	p15A	Kan	this work	JBx_092821
pLane-EilR	N/A	N/A	colE1	Kan	this work	JBx_092825
pJC575	P_{JEx1}	*rfp*	colE1, R2K	Kan	this work	JBx_045211
pJC577	P_{JExA1}	*rfp*	colE1, R2K	Kan	this work	JBx_048484
pJC578	P_{JExA2}	*rfp*	colE1, R2K	Kan	this work	JBx_048485
pJC579	P_{JExL}	*rfp*	colE1, R2K	Kan	this work	JBx_048486
pJC580	P_{JExD}	*rfp*	colE1, R2K	Kan	this work	JBx_048487
pJC581	P_{JExH1}	*rfp*	colE1, R2K	Kan	this work	JBx_048488
pJC582	P_{JExH2}	*rfp*	colE1, R2K	Kan	this work	JBx_048489
pJC583	$P_{LtetO-1}$	*rfp*	colE1, R2K	Kan	this work	JBx_048490
pJC586	$P_{LtetO-1}$	N/A	colE1, R2K	Kan	this work	JBx_048493
pJC681	P_{JExH1}	*pleC*	colE1, R2K	Kan	this work	JBx_089877

* Based on bicistronic RFP reporter[12] with the divergently oriented weak constitutive promoter aPFAB254

Supplementary Table 5 | List of primers used in this work.

a **Primers for randomized promoter library**

eilO-pFAB_random_for	**tgtccaacT**ANNNTgtgtggagggcccaagttcac*
eilO-pFAB_random_rev	**cgtgtccaa**NNNCAAgttatgcagcaacgactcatagaaagc*

* eil-operator marked in bold -35 and -10 sites in upper case

b **insertion of intermediate phage promoters containing truncated *eilOc* into pTR_eilO1t**

**promoter sequence incl. operator (variable part of gBlock)**	**resulting plasmid***
aaaatttatcaaaaagagtattgacttggacacgtgtccaactgatacttacagccatcgagagggacacggcga	pPJExA1t
aaaatttatcaaaaagagtattgacttaaagttggacacgtgtgatacttacagccatcgagagggacacggcga	pPJExA2t
aaaaaactgcaaaaaaatagtttgacaggacacgtgtccaactttaagatgtacccagttcgatgagagcgataac	pPJExDt
ataatttttaaaaaaattcatttgctaaagttggacacgtgtcctataatatacttcataaattgataaacaaaaa	pPJExH1t
ataatttttaaaaaaattcatttgctaggacacgtgtccaactttataatatacttcataaattgataaacaaaaa	pPJExH2t
ataaattatctctggcggtgttgacaaagttggacacgtgtccgatactgagcacatcagcaggacgcactgacc	pPJExLt

* intermediate plasmids for construction of final promoter versions in (c)

c **addition of full length *eilOc* at transcriptional start**

**forward primer name**	**forward primer sequence**
Phage_Low_for	gtgtccaactttgaattcaaaagatcttt
Phage_High_for	gtgtccaactttgaattcaaaagatcttttaagaaggag
PEilO1t_for	gtgtccaactttgaattcaaaagatcttttaagaaggagatatacatatg

**reverse primer name**	**reverse primer sequence**	**F-primer used**	**template plasmid**	**resulting promoter**
PJExA1t-rev	gtgtccaactttggctgtaagtatcagttgg	Phage_High_for	pPJExA1t	PJExA1
PJExA2t-rev	gtgtccaactttggctgtaagtatcacacg	Phage_High_for	pPJExA2t	PJExA2
PJExDt-rev	gtgtccaactttgggtacatcttaaagtt	Phage_Low_for	pPJExDt	PJExD
PJExH1t-rev	gtgtccaactttgaagtatattataggaca	Phage_Low_for	pPJExH1t	PJExH1
PJExH2t-rev	gtgtccaactttgaagtatattataaagtt	Phage_Low_for	pPJExH2t	PJExH2
PJExLt-rev	gtgtccaactttgtgctcagtatcggaca	Phage_Low_for	pPJExLt	PJExL
PEilO1t-rev	gtgtccaactttccacacatcatagttggac	PEilO1t_for	pTR_eilO1t	PJEx1

Supplementary Table 6 | Oligonucleotides used for the electrophoretic mobility shift assay: The native operator sequences *eilO1* and *eilO2* located on the *E. lignolyticus eilAR* intergenic region; the full-length and half-length consensus operator *eilOc*; and a random DNA sequence. Highlighted letters delineate the operator sequence.

**eilO1**	A	A	**A**	**A**	**A**	**G**	**C**	**T**	**G**	**G**	**A**	**C**	**A**	A	G	**T**	**G**	**T**	**T**	**C**	**A**	**A**	**C**	**T**	**T**	**T**	C	C
**eilO2**	C	G	**C**	**A**	**A**	**A**	**C**	**T**	**G**	**G**	**A**	**C**	**G**	G	A	**T**	**G**	**T**	**C**	**C**	**A**	**G**	**C**	**T**	**T**	**T**	G	T
**eilOc**	A	A	**A**	**A**	**A**	**G**	**T**	**T**	**G**	**G**	**A**	**C**	**A**	C	G	**T**	**G**	**T**	**C**	**C**	**A**	**A**	**C**	**T**	**T**	**T**	C	C
**1/2 eilOc**	A	A	**A**	**A**	**A**	**G**	**T**	**T**	**G**	**G**	**A**	**C**	**A**	C	C	A	G	G	A	C	T	C	C	T	C	C	C	C
**random**	A	A	G	T	T	A	C	C	G	T	T	A	C	C	C	A	G	G	A	C	T	C	C	T	C	C	C	C

Supplementary References

1. Ruegg, T. L. *et al.* An auto-inducible mechanism for ionic liquid resistance in microbial biofuel production. *Nat. Commun.* **5,** 3490 (2014).
2. Lee, T. S. *et al.* BglBrick vectors and datasheets: A synthetic biology platform for gene expression. *J Biol Eng* **5,** 12 (2011).
3. Yamasaki, S. *et al.* The crystal structure of multidrug-resistance regulator RamR with multiple drugs. *Nat. Commun.* **4,** 1–7 (2013).
4. Cupples, C. G. & Miller, J. H. A set of lacZ mutations in *Escherichia coli* that allow rapid detection of specific frameshift mutations. *Proc. Natl. Acad. Sci.* **86,** 5345–5349 (1989).
5. Song, L. Y. *et al.* Mutational consequences of ciprofloxacin in *Escherichia coli*. *Antimicrob. Agents Chemother.* **60,** 6165–6172 (2016).
6. Haldimann, A. & Wanner, B. L. Conditional-replication, integration, excision, and retrieval plasmid-host systems for gene structure-function studies of bacteria. *J. Bacteriol.* **183,** 6384–6393 (2001).
7. Fields, A. T. *et al.* The conserved polarity factor PodJ1 impacts multiple cell envelope-associated functions in *Sinorhizobium meliloti*. *Mol. Microbiol.* **84,** 892–920 (2012).
8. Studier, F. W. & Moffatt, B. A. Use of bacteriophage T7 RNA polymerase to direct selective high-level expression of cloned genes. *J. Mol. Biol.* **189,** 113–130 (1986).
9. Guzman, L. M., Belin, D., Carson, M. J. & Beckwith, J. Tight regulation, modulation, and high-level expression by vectors containing the arabinose P(BAD) promoter. *J. Bacteriol.* **177,** 4121–4130 (1995).
10. Haldimann, A., Daniels, L. L. & Wanner, B. L. Use of new methods for construction of tightly regulated arabinose and rhamnose promoter fusions in studies of the *Escherichia coli* phosphate regulon. *J. Bacteriol.* **180,** 1277–1286 (1998).
11. Balzer, S. *et al.* A comparative analysis of the properties of regulated promoter systems commonly used for recombinant gene expression in *Escherichia coli*. *Microb. Cell Fact.* **12,** 26 (2013).
12. Skerra, A. Use of the tetracycline promoter for the tightly regulated production of a murine antibody fragment in *Escherichia coli*. *Gene* **151,** 131–135 (1994).
13. Choi, Y. J. *et al.* Novel, versatile, and tightly regulated expression system for *Escherichia coli* strains. *Appl. Environ. Microbiol.* **76,** 5058–5066 (2010).
